# Leukocyte Immunoglobulin-Like Receptor 1-Expressing Human Natural Killer Cell Subsets Differentially Recognize Isolates of Human Cytomegalovirus through the Viral Major Histocompatibility Complex Class I Homolog UL18

**DOI:** 10.1128/JVI.02614-15

**Published:** 2016-02-26

**Authors:** Kevin C. Chen, Richard J. Stanton, Jareer J. Banat, Mark R. Wills

**Affiliations:** a Department of Medicine, University of Cambridge, Cambridge, Cambridgeshire, United Kingdom; b Cardiff Institute of Infection and Immunity, Cardiff, United Kingdom; c Medical Education Centre, Altnagelvin Hospital, City of Derry, United Kingdom

## Abstract

Immune responses of natural killer (NK) cell are controlled by the balance between activating and inhibitory receptors, but the expression of these receptors varies between cells within an individual. Although NK cells are a component of the innate immune system, particular NK cell subsets expressing Ly49H are positively selected and increase in frequency in response to cytomegalovirus infection in mice. Recent evidence suggests that in humans certain NK subsets also have an increased frequency in the blood of human cytomegalovirus (HCMV)-infected individuals. However, whether these subsets differ in their capacity of direct control of HCMV-infected cells remains unclear. In this study, we developed a novel *in vitro* assay to assess whether human NK cell subsets have differential abilities to inhibit HCMV growth and dissemination. NK cells expressing or lacking NKG2C did not display any differences in controlling viral dissemination. However, when *in vitro*-expanded NK cells were used, cells expressing or lacking the inhibitory receptor leukocyte immunoglobulin-like receptor 1 (LIR1) were differentially able to control dissemination. Surprisingly, the ability of LIR1^+^ NK cells to control virus spread differed between HCMV viral strains, and this phenomenon was dependent on amino acid sequences within the viral ligand UL18. Together, the results here outline an *in vitro* technique to compare the long-term immune responses of different human NK cell subsets and suggest, for the first time, that phenotypically defined human NK cell subsets may differentially recognize HCMV infections.

**IMPORTANCE** HCMV infection is ubiquitous in most populations; it is not cleared by the host after primary infection but persists for life. The innate and adaptive immune systems control the spread of virus, for which natural killer (NK) cells play a pivotal role. NK cells can respond to HCMV infection by rapid, short-term, nonspecific innate responses, but evidence from murine studies suggested that NK cells may display long-term, memory-like responses to murine cytomegalovirus infection. In this study, we developed a new assay that examines human NK cell subsets that have been suggested to play a long-term memory-like response to HCMV infection. We show that changes in an HCMV viral protein that interacts with an NK cell receptor can change the ability of NK cell subsets to control HCMV while the acquisition of another receptor has no effect on virus control.

## INTRODUCTION

Following primary human cytomegalovirus (HCMV) infection, lytic viral replication is controlled by the host immune response, which includes humoral (1, 2), innate (3, 4), and adaptive (5–7) cellular immune responses. Despite this robust immune response, the virus is still able to establish latency in myeloid progenitor cells (8, 9). Virus can reactivate when these cells differentiate to mature dendritic cells, and as such the virus is able to persist for the lifetime of the host. Primary infection of healthy immunocompetent individuals is most often asymptomatic, but the virus can cause severe diseases in immunocompromised transplant patients, immunocompromised patients with AIDS, and the immune immature, particularly following *in utero* infection (10–14).

Natural killer (NK) cells are defined as a component of the innate immune system, as they do not undergo somatic DNA rearrangements in order to express highly diverse antigen receptors in the same manner as B and T cells do (15). Instead, NK cells express a wide variety of activating and/or inhibitory receptors that are able to bind cellular ligands, some of which are normally expressed while others are induced by infection or transformation (reviewed in reference 16). The balance between activating and inhibitory signals determines if an NK cell is activated and exerts an effector function or not. NK cells are implicated in control of herpesvirus infections, since individuals with rare NK cell defects have been shown to have difficulty controlling multiple different herpesvirus infections, including HCMV (17, 18).

In order to avoid this NK cell response, HCMV encodes multiple proteins that modulate NK cell recognition of infected cells (19, 20). These NK evasion functions act by preventing cellular ligands binding to activating NK cell receptors (UL16, UL141, UL142, US18, US20, US9 [21–27], and miR-UL112 [21]), by expressing proteins that engage inhibitory NK cell receptors (UL18 [28], UL40 [20, 29]), and UL83 [30]), and by modifying the structure of the immune synapse (UL135 [31]).

However, NK cells are not homogeneous; instead, numerous different NK cell subsets exist within a given individual, since individual activating and inhibitory NK receptors are independently expressed in varied combinations on different cells. Murine studies have shown that the interaction between murine cytomegalovirus (MCMV) protein m157 and the activating Ly49H receptor on murine NK cells leads to direct activation of NK cells and the control of MCMV disease (32). In contrast, the only known example of direct NK cell receptor binding with HCMV protein is the interaction of leukocyte immunoglobulin-like receptor 1 (LIR1, now commonly known as LILRB1), an inhibitory receptor that normally binds to human major histocompatibility complex class I (MHC-I) molecules, with the HCMV protein UL18, a viral homolog of cellular MHC-I-like molecules (33, 34). Early work on the UL18 protein from HCMV strain AD169 suggested that it could enhance cytotoxic killing by an NK cell line against an Epstein-Barr virus (EBV)-infected 293 cell line target in chromium release assays (35); however, these experiments did not consider the level of expression of LIR1 on NK cells. Subsequently, Prod'homme et al. (28) showed that the UL18 protein from HCMV strain AD169 actually lowered the short-term cytotoxic responses of NK cells, but only if they expressed LIR1 (LIR1^+^), leading to the conclusion that UL18 was an immunoregulatory protein that inhibited NK cells from clearing HCMV lytically infected cells (28).

The activating C-type lectin receptor CD94/NKG2C, which normally binds to human HLA-E, can also bind with HCMV UL18 protein but with 1,000-fold-weaker affinity than LIR1 (36). NKG2C^+^ NK cells have been shown to be preferentially expanded in HCMV-seropositive individuals (37–42). *In vitro* experiments demonstrated that HCMV can induce expansion of CD94/NKG2C^+^ NK cells (43), and these cells show enhanced cytotoxic responses against HCMV-infected cells in the presence of HCMV-specific antibodies (44). Except UL18, no other HCMV viral ligand has been shown to bind directly with NKG2C/CD94. Moreover, although these reports support a strong correlation between an increase in NKG2C^+^ NK cells and HCMV serostatus (reviewed in reference 16), no report to date has determined whether the expression of NKG2C on NK cells, in the absence of HCMV-seropositive donor serum (44), leads to better control of virus.

So far, the interpretation of the role of LIR1 and NKG2C receptors has focused mainly on NK cell degranulation and cytotoxicity effector functions over short-term (4- to 6-h) coculturing of NK cells with infected target cells. However, NK cells also secrete inflammatory cytokines and can replicate after activation, and these can also influence virus replication (16). Thus, longer-term assessment of antiviral activity of NK cells and particularly of NK cell subpopulations would be a valuable method to further understand the interaction of NK cells with HCMV-infected cells.

We have recently developed and utilized such a viral dissemination assay (VDA) to examine the antiviral activity of HCMV-specific T cells (45). In this study, we have used a VDA in conjunction with coculture of different NK cell subsets in order to compare their abilities to inhibit HCMV dissemination. *In vitro*-expanded LIR1^+^ NK cells controlled the spread of laboratory HCMV strain AD169 less effectively than did LIR1-nonexpressing (LIR1^−^) NK cells, in good agreement with the NK cell effector function analyses by Prod'homme et al. (28). However, when low-passage-number/clinical strains TB40/e and Merlin were used in the VDA, this was not the case. In fact, LIR1^+^ NK cells displayed stronger control of virus spreading than did LIR1^−^ NK cells, and this was observed in multiple different donors. Using the published crystal structure of the LIR1-UL18/B_2_m complex to inform the generation of specific HCMV mutants, we identified three amino acids in the viral UL18 protein that were responsible for this phenomenon. Furthermore, using NK cell subsets expressing LIR1 and NKG2C, we demonstrated that LIR1, rather than NKG2C, plays the dominant role in influencing the long-term responses of LIR1^+^ NK cells during HCMV dissemination.

## MATERIALS AND METHODS

### Donor sample collection and isolation.

Heparinized peripheral blood was collected from healthy donors. HCMV serostatus was determined using an IgG enzyme-linked immunosorbent assay (Trinity Biotech, Didcot, United Kingdom). Ten HCMV-seronegative and five HCMV-seropositive donors were included in this study. Ethical approval involving donor peripheral blood was obtained from the Addenbrookes National Health Service Hospital Trust institutional review board (Cambridge Research Ethics Committee) for this study. Informed written consent was obtained from all recipients in accordance with the Declaration of Helsinki (LREC 97/092).

### Cells and viruses.

The human foreskin fibroblast (HFF) cell line was obtained from a commercial company (Invitrogen, Paisley, United Kingdom) and was cultured in MEM-10, consisting of Eagle's minimal essential medium (EMEM; Life Technologies) supplemented with 10% fetal calf serum (PAA, Linz, Austria), 100,000 IU/ml penicillin (Life Technologies), and 100 mg/ml streptomycin (Life Technologies). The strains of HCMV used in these studies were AD169, AD169 with the UL18 open reading frame (ORF) deleted (AD169-ΔUL18) (46), strain Merlin containing a green fluorescent protein (GFP)-UL32 fusion protein (Merlin) (47), Merlin-GFP-UL32 with UL18 ORF deletion (Merlin-ΔUL18), and TB40/e-GFP-UL32 (TB40/e; kind gift of Christian Sinzger). In addition, the UL18 sequence-modified virus strains (AD169-UL18Merlin and Merlin-UL18AD169) were generated by recombineering as previously described (48) using the primers listed in Table 1. The method used to generate the mutant virus isolates does not lead to the loss of US2-US6 HCMV genes.

**TABLE 1:** Primers used for UL18 mutant virus generation^*a*^

Primer name	Sequence
Merlin-F	GACAACAGAGCTGAAGCATTCTGTACATCTTACGGGTTCTTTCCAGGGGAAATTAATATTACTTTTATCCATTACGGTCCTGTGACGGAAGATCACTTCG
Merlin-R	AAAAGATGGCTACGTAACATCCCTGATGGAAAGTCCCATCGAAGGTGGGAAGTAGCGGATTGCATTGAGGCTCGCTATCCTGAGGTTCTTATGGCTCTTG
AD169-F	GACAACAGAGCTGAAGCATTCTGTACATCTTACGGGTTCTTTCCAGGGGAAATTAATATTACTTTTATTCATTACGGTCCTGTGACGGAAGATCACTTCG
AD169-R	AAAAGATGGCTACGTAACATCCCTGATGGAAAGTCCCATCCAAGGTGGGAAGTAGCGGATTGCATTGAGGCTCGCTATCCTGAGGTTCTTATGGCTCTTG
Primer-F	AAACAAAACGTACATCGACGGTAA
Primer-R	AGCAAAGCGCATAAAAGCAGG

a Sequences used to generate AD169-UL18 Merlin mutant virus are designated Merlin-F (F, forward primer) and Merlin-R (R, reverse primer); those used to generate Merlin-UL18AD mutant virus are designated AD169-F and AD169-R. Primer-F and Primer-R were used to amplify the a3 domain of HCMV UL18 protein in a standard PCR.

### Sequencing of HCMV UL18 ORF.

DNA was extracted from HCMV-infected fibroblasts using the DNeasy-blood and tissue kit (Qiagen). A 30-cycle PCR was performed to amplify viral UL18 ORF using the forward primers shown in Table 1 and under conditions previously described (49). Samples were sequenced by the Source Bioscience Sequencing Team, Cambridge, United Kingdom.

### Preparation of *in vitro*-expanded NK cells.

Fresh peripheral venous blood was obtained by venipuncture, performed by a trained phlebotomist. Peripheral blood mononuclear cells (PBMC) were isolated from fresh peripheral venous blood by Ficoll Hypaque density gradient centrifugation (Axis-Shield, Oslo, Norway) as previously described (50). NK cells were purified from PBMC, using the EasySep-Human NK cell enrichment kit (StemCell Technologies). *In vitro*-expanded, activated NK cell lines (referred to here as *in vitro*-expanded NK cells) were then generated from *ex vivo* NK cells by coculturing with irradiated allogeneic Epstein-Barr virus (EBV)-transformed B cell lines and irradiated autologous PBMC using methods previously described (24). The cell lines were cultured in RPMI-10 (described earlier) with 25 IU/ml interleukin-2 (IL-2; National Institute for Biological Standards and Controls) replenished every 5 days.

### Preparation of sorted NK cell subsets.

*In vitro*-expanded NK cell lines were stained with mouse anti-human CD56-Pacific Blue (PB) or fluorescein isothiocyanate (FITC; eBioscience, United Kingdom), CD3-PerCP/Cy5.5 (Biolegend UK), LIR1/CD85j-phycoerythrin (PE) (Biolegend UK), and NKG2C-allophycocyanin (APC) (Biolegend UK) antibodies. CD56^+^ CD3^−^ NK cells were sorted into different populations using a FACSJazz cell sorter (where FACS stands for fluorescence-activated cell sorter) running FACS DIVA software (Becton Dickinson, United Kingdom).

### CD107a degranulation assays.

K562 cells (1 × 10^6^) or HCMV-infected fibroblasts (subjected to overnight infection with TB40/e at a multiplicity of infection [MOI] of 5) were cocultured with *in vitro*-expanded NK cells at a ratio of 1:1 in 50 μl RPMI-10 and incubated at 37°C and 5% CO_2_. Monensin (BioLegend) was added at a 1:1,000 dilution after 1 h of coincubation and further incubated for 4 h at 37°C and 5% CO_2_. Cells were washed in phosphate-buffered saline (PBS) before staining with anti-human CD56 (APC), CD3-FITC, and CD107a PerCP/Cy5.5 antibodies (Biolegend UK) before being analyzed by flow cytometry using FACSCalibur (BD) running CellQuest software (BD). Results were analyzed using FlowJo 7.6 (Tree Star Inc., Ashland, OR, USA).

### CCL4 cytokine ELISAs.

*In vitro*-expanded NK cells were cocultured overnight with K562 cells or HCMV-infected fibroblasts (overnight infection with TB40/e at an MOI of 5) using the method described earlier for the CD107a assays, but without monensin. The cocultures were left for 12 h before the supernatant was harvested, and the CCL4 cytokine concentration was measured using an enzyme-linked immunosorbent assay (ELISA) kit (R&D systems).

### *In vitro* viral dissemination assay.

The ability of NK cell subsets (sorted based on differences in specific receptor expression) to control the spread of HCMV *in vitro* was measured. Allogeneic HFF cells were seeded in 24-well flat-bottom culture plates (LifeSciences, United Kingdom) to be 80 to 90% confluent when they were infected with virus at an MOI of 0.1 overnight. Rested *in vitro*-expanded NK cells were harvested, washed, stained, sorted, and resuspended in MEM-10 and then added to the infected fibroblasts at an NK cell-to-fibroblast ratio of 1.25:1, 0.625:1, or 0.3125:1 in 1 ml MEM-10 and incubated at 37°C and 5% CO_2_. Assessment of viral dissemination was performed at 9 days postincubation. GFP expression (TB40/e and Merlin) was detected by either fluorescence microscopy or flow cytometry. When HCMV AD169 was used, fibroblasts were stained intracellularly with anti-CMV immunoelectrophoresis (IE)-Alexa Fluor488 antibody (Millipore) using the Cell Fixation/Permeabilization kit (An-Der-Grub BioResearch). Fibroblasts were fixed with a 2% paraformaldehyde–PBS solution and analyzed using flow cytometry as described earlier.

Viral spread in each well was determined as a percentage of control wells lacking NK cells, using the following equation: ([Experimental % of infected cells − background % of HFF-only control]/[% of infected HFF control without NK cells − background % of HFF-only control]) × 100.

### Phylogenetic tree.

BioEdit Sequencing Alignment Editor was used for sequence analysis. The evolutionary phylogenetic trees were computed using Molecular Evolutionary Genetics Analysis (MEGA).

### Statistics.

Statistical analysis was performed using GraphPad Prism version 4.00 for Windows (GraphPad Software, San Diego, CA). Probabilities were calculated with one-way or two-way analysis of variance (ANOVA) paired Friedman test in the viral dissemination assay, assuming not-repeated measures. Standard *t* test analysis was used to analyze the cytokine CCL4 release assay and CD107a degranulation assay. Results with *P* values of <0.05 were considered significant.

## RESULTS

### Control of different strains of HCMV dissemination by *in vitro*-expanded primary NK cell lines.

We have previously developed an assay to measure T cell-mediated antiviral activity based on the inhibition of HCMV dissemination through a permissive fibroblast monolayer (51, 52). In addition, a focal expression assay that is similar in concept to our viral dissemination assay (VDA) has recently been described by others (52) and used to examine anti-HCMV activity by NK cells (45). We wished to use our VDA to study the antiviral activity of different NK cell subsets, as defined by specific cell surface markers.

**FIG 1:**
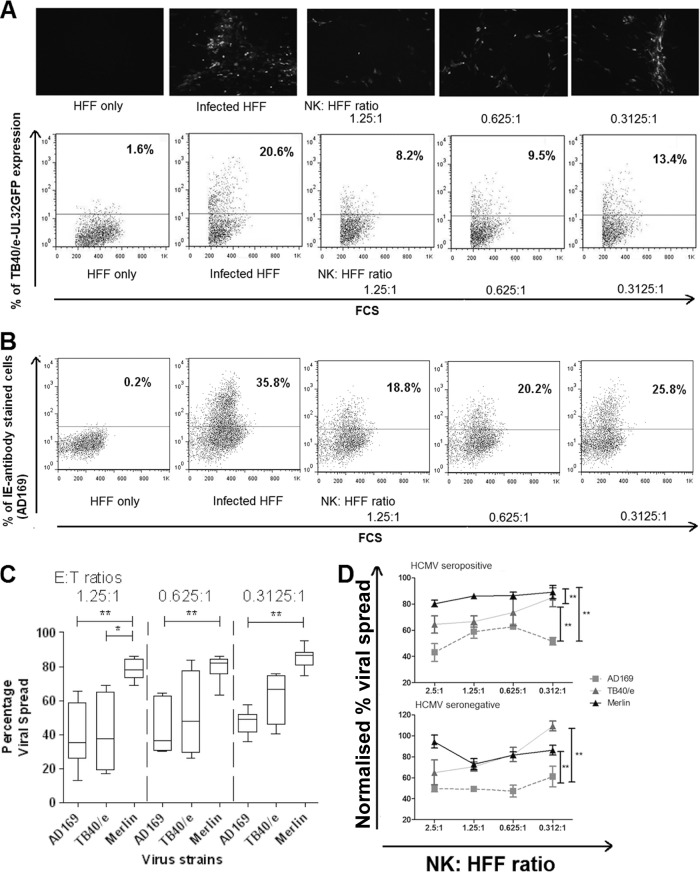
Establishment of viral dissemination assay. (A) Human fibroblasts (HFFs) infected with HCMV strain TB40/e UL32-GFP at an MOI of 0.1 and *in vitro*-expanded NK cells were cocultured for 9 days at various effector-to-target (E/T) ratios, starting from 1.25:1 to 0.3125:1, at 37°C and 5% CO_2_. NK cells were then washed off, and the HFFs were observed by fluorescence microscopy (top panel) or following trypsinization analyzed by flow cytometry (bottom panel). Representative dot plots show the results from uninfected and infected controls and the change percentage of fluorescent cells following coincubation with different ratios of NK cells to HFFs. (B) The assay was also performed using HFFs infected with untagged AD169 at an MOI of 0.1. The cells were stained intracellularly with anti-IE-Alexafluor488 antibodies before being analyzed by flow cytometry. (C) Summary results of dissemination assays on NK cells derived from 7 donors using AD169-infected HFFs, from 4 donors using TB40/e-infected HFFs, and from 9 donors using Merlin-infected HFFs. The MOI used was 0.1, and various effector-to-target (E/T) ratios from 1.25:1 to 0.3125:1 were used. At the end of the assay, TB40/e-infected or Merlin-infected HFFs were analyzed by flow cytometry without additional staining, while AD169-infected HFFs were stained with anti-IE-Alexa Fluor 488 antibody before analysis by flow cytometry. Data were analyzed by one-way ANOVA, and significant results (*, *P* < 0.05; **, *P* < 0.01) are indicated. (D) Three separate dissemination assays using HCMV strain AD169, TB40/e, or Merlin at an MOI of 0.1 were conducted using *in vitro*-expanded NK cells from either a seropositive donor (donor 319) or a seronegative donor (donor 401). The data were normalized according to the uninfected and infected controls. The black triangles are results from Merlin-infected HFF; gray triangles are TB40/e-infected HFF; and gray squares are AD169-infected HFF. Each data point represents 3 independent readouts, and error bars represent the standard errors of the means (SEM). Data were analyzed by two-way ANOVA, and significant results (*, *P* < 0.05; **, *P* < 0.01) are indicated.

In order to validate this approach, we used the VDA against different strains of HCMV to determine if *in vitro*-expanded NK cells were able to control the spread of high-passage-number laboratory-adapted (AD169) and low-passage-number isolates of HCMV TB40/e and Merlin. The results clearly show that NK cells were able to prevent viral spread in an effector-to-target ratio (E/T)-dependent fashion. This was visualized by fluorescence microscopy and quantified by flow cytometry, either by GFP if the virus expressed GFP (Fig. 1A) or by anti-HCMV IE antigen staining with a fluorescent antibody if it did not (Fig. 1B).

High-passage-number strains such as AD169 lack multiple immune evasion genes. This renders AD169-infected cells more susceptible to NK cell-mediated lysis than low-passage-number HCMV isolates such as TB40/e or Merlin in short-term cytotoxicity assays (53, 54). In order to compare VDA results between different strains of virus, data for each virus were normalized against their own positive (HCMV-infected HFFs without NK cells) and negative (noninfected HFFs) controls. The positive control represents the maximal spread of the virus over the course of the assay, while the negative control is the background fluorescence.

Across multiple donors, it was clear that the virus spread of AD169 (*n =* 4) was significantly reduced compared to those of Merlin (*n =* 9) and TB40/e (*n =* 7), implying that polyclonally activated NK cells were significantly more efficient at controlling the spread of AD169 (Fig. 1C). In addition, TB40/e was controlled less well than Merlin at the highest E/T ratio of 1.25:1. A similar trend was evident at the lower ratios, although this was not statistically significant (Fig. 1C). Importantly, NK cells still exerted some degree of control over Merlin, as viral spread did not reach 100% for any of the E/T ratios tested (Fig. 1C). We also performed VDAs using *in vitro*-expanded NK cells derived from either an HCMV-seropositive or -seronegative donor (Fig. 1D). No major differences were seen between the polyclonal NK cells from these donors, with cells from both being significantly more efficient at controlling the spread of AD169 than either TB40/e or Merlin.

Taken together, these results demonstrate that, using this NK cell VDA, a low-passage-number isolate of HCMV (Merlin) was resistant to NK cell-mediated control, while an isolate lacking NK immune evasion genes (AD169) was less resistant. Despite low-passage-number strains being more resistant to NK cell control, polyclonal NK cells did still exert some degree of control, indicating that this assay represented a useful method to study the antiviral properties of different subsets of NK cells as well as the ability of different HCMV isolates to affect NK cell recognition.

### Comparison of LIR1^+^ and LIR1^−^ NK cell control of HCMV dissemination.

The VDA was able to identify differences in the ability of NK cells to control different HCMV strains; we next wanted to use the assay to determine if certain NK cell subsets, as defined by surface expression of particular phenotypic markers, were more efficient at controlling HCMV infection. The HCMV MHC-I homolog UL18 (specifically from strain AD169) has already been shown to decrease direct NK cell cytotoxic responses, as it is able to bind the inhibitory receptor LIR1 present on some NK cell subsets (28). As such, AD169 should be less well controlled by LIR1^+^ NK cells (which would be inhibited by HCMV UL18 protein expression) than by LIR1^−^ NK cells (which would not be inhibited by UL18 [28]).

**FIG 2:**
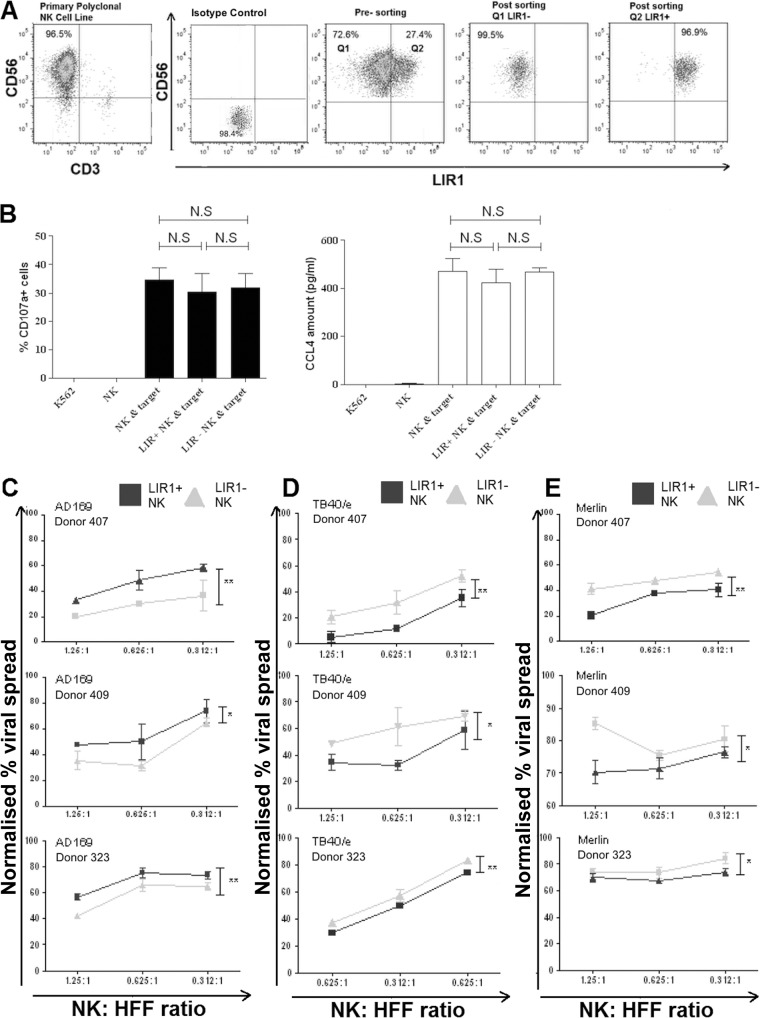
NK cells expressing LIR1 have effector cellular functions similar to those of LIR1-nonexpressing NK cells but are more able to control viral dissemination of HCMV strains TB40/e and Merlin. (A) *In vitro*-expanded NK cells were stained with anti-CD3, -CD56, and -LIR1 antibodies and sorted by flow cytometry. CD56^+^ CD3^−^ NK cells were first collected (NK) before further sorting into LIR1^+^ (LIR1^+^ NK) and LIR1^−^ (LIR1^−^ NK) subsets based on LIR1 expression. Representative dot plots of the NK cells before and after sorting are shown. (B) NK, LIR1^+^ NK and LIR1^−^ NK cells were cocultured with K562 target cells. The NK cell-to-target ratio is 1:1. K562 cells and NK cells only are the controls. After culturing for 5 h, the percentages of CD107a^+^ cells were measured by flow cytometry. CCL4 concentration is quantified using ELISA after culturing overnight. Each data point represents the mean value of 3 repeats, and error bars represent SEM. The experiment was preformed using three different donors (*n =* 3), and the results showed the average values analyzed by the Student *t* test. Nonsignificant results (NS; *P* > 0.05) are indicated. (C) LIR1^+^ and LIR1^−^ NK cell subsets were cocultured with human HFFs infected with HCMV strain AD169, TB40/e, or Merlin at an MOI of 0.1 in a viral dissemination assay. The NK cell-to-target ratios are 1.25:1, 0.625:1, and 0.312:1. The data were normalized according to the uninfected and infected controls. The gray triangles are results from LIR1^−^ NK cells, and black squares are the results from LIR^+^ NK cells. Each data point represents 3 independent readouts, and error bars represent SEM. In total, each assay was repeated three times (*n =* 3) using NK cells from three different donors. Data were analyzed by two-way ANOVA, and significant results (*, *P* < 0.05; **, *P* < 0.01) are indicated.

Primary *in vitro*-expanded NK cell lines were generated from different donors; NK cells were sorted as CD3^−^ CD56^+^ cells before sorting into subsets based on their LIR1 expression, achieving >95% purity after sorting (Fig. 2A). To ensure that the subsets maintained their effector functions, postsorting cytotoxicity and cytokine secretion against the classic K562 target cells were examined (55). NK cell cytotoxicity was determined by CD107a degranulation assay, and cytokine production was assessed by measuring CCL4 production, one of the earliest inflammatory cytokines produced following NK cell activation (56). While the NK cell lines were capable of generating gamma interferon (IFN-γ) against K562 target cells, the response was modest, between 20 and 30 pg/ml. The CCL4 response was more substantial at 400 pg/ml and was also elicited by HCMV-infected target cells; therefore, we measured CCL4 for all the subsets, as it provided a better dynamic range in order to determine if cell sorting had caused differences in the sorted subsets.

Both LIR1^+^ and LIR1^−^ subsets had a cytotoxic response and level of CCL4 secretion similar to those of the unsorted NK cell line from which they were derived (Fig. 2B). Thus, NK cells maintain their cellular functions after sorting, and this also suggests that engagement of anti-LIR1 antibodies did not cause inhibition of LIR1^+^ NK cell effector functions.

The LIR1^+^ and LIR1^−^ NK cell subsets were then used in a VDA against AD169-infected HFFs. As predicted, the results show that there was a greater percentage of viral spread in the presence of LIR1^+^ NK cells than LIR1^−^ NK cells (Fig. 2C). The VDA was also performed using HFFs infected with the low-passage-number HCMV strains TB40/e and Merlin. Surprisingly, different results were obtained. Against these strains, LIR1^+^ NK cells demonstrated more efficient virus control than LIR1^−^ NK cells (Fig. 2D and E). The same results were observed for three independent donor NK cell lines tested (Fig. 2C to E). Thus, differences between HCMV strains can affect the ability of NK cell subsets to control virus spread.

### Deletion of HCMV UL18 ORF abolishes the differential controls mediated by LIR1^+^ and LIR1^−^ NK cells.

We next investigated whether the above phenomenon (Fig. 2C to E) was caused by the viral UL18 protein. If it was due to the interaction of UL18 on HCMV-infected cells with LIR1 on NK cells, the difference should be negated if a virus with UL18 deleted or a blocking antibody to LIR1 were used in these assays. While LIR1-blocking antibodies are available, they could deliver inhibitory signals through LIR1, and we were concerned that the antibody concentrations could be maintained over the long period of this assay; therefore, we determined the involvement of UL18 by using UL18 deletion mutants of both AD169 and Merlin.

**FIG 3:**
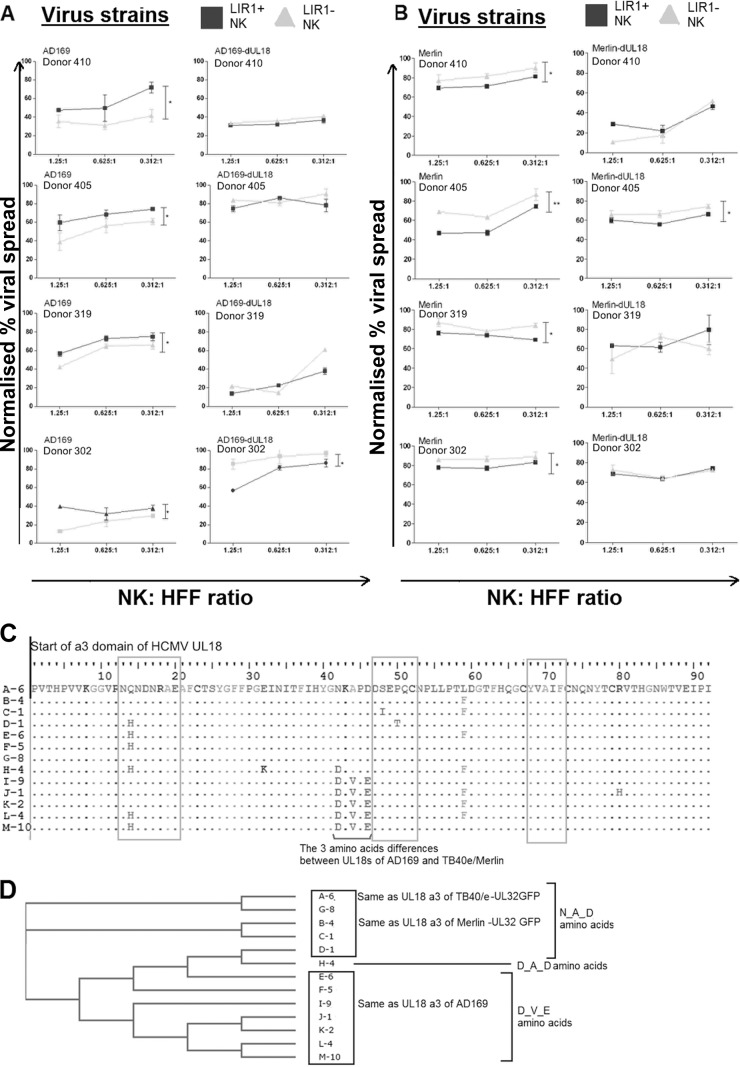
UL18 proteins from different HCMV strains influence the control of virus dissemination by LIR1^+^ NK cells. (A) Sixty-seven sequences of HCMV UL18 proteins were identified from the NCBI protein database (www.ncbi.nlm.nih.gov). Six sequences had a truncation and are not included in the alignment. Thirteen unique amino acid sequences of the α3 region of UL18 were identified. Each unique sequence is shown only once in the alignment and is represented by a unique letter. The number following the letter in the sequence name represents the number of times the sequence has appeared in the database. Gray boxes indicate the sites interacting with LIR1 as suggested by the crystal structure (57), while the black line indicates the key differences between the UL18 of AD169 and Merlin strains. (B) Results of the neighbor-phylogenic tree analysis showing the relationship between the sequences based on the protein α3 region. Twenty virus strains have an NKAPED sequence, 37 have DKVPED, and 4 have DKAPDD. (C) NK cells from four different donors (*n =* 4) were sorted based on LIR1 expression as previously described before being cocultured in a viral dissemination assay with HFFs infected with HCMV strain AD169 or AD169-ΔUL18 at an MOI of 0.1. Infected cells were stained with IE-Alexa Fluor 488 antibodies before analysis using flow cytometry. (D) The viral dissemination assay was repeated with NK cells from four different donors (*n =* 4) sorted based on LIR1 expression and cocultured with HFFs infected with HCMV strain Merlin or Merlin-ΔUL18 at an MOI of 0.1. Infected HFFs were analyzed on the basis of UL32-GFP fluorescence. In panels C and D, the gray triangles are results from LIR1^−^ NK cells; black squares are the results from LIR^+^ NK cells. The NK cell-to-target ratios range from 1.25:1, 0.625:1, and 0.312:1. Each data point represents 3 independent readouts. The data were analyzed using two-way ANOVA, error bars represent SEM, and significant results (*, *P* < 0.05; **, *P* < 0.01) are indicated.

VDAs were conducted using HCMV AD169-ΔUL18 and Merlin-ΔUL18 viruses as well as their parental strains. As before, LIR1^+^ NK cells were less able to control the spread of AD169 than were LIR1^−^ NK cells; however, when AD169-ΔUL18 was used, this difference was eliminated (Fig. 3A). In donor 410, the LIR1^−^ NK cells exhibit the same level of viral control for both AD169 and AD169-ΔUL18 viruses; however, the major change occurs with LIR1^+^ NK cells, which exert better control over AD169-ΔUL18 virus than AD169, in agreement with results from the other independent donors. In donor 319, we noted a discrepancy in that both LIR1^+^ and LIR1^−^ cells exerted greater control when UL18 had been deleted in this experiment. However, this seems to be donor specific (donor-to-donor variation) rather than virus specific, as it does not occur in other donors (donor 405, for example), indicating that it is unlikely to be due to additional changes in the AD169-ΔUL18 strain. Nevertheless, although with donor 319 the AD169-ΔUL18 strain had a lower spread than expected in the presence of both LIR1^+^ and LIR1^−^ NK cells, the two subsets did maintain the same pattern as the other donors (i.e., there were no significant differences between the subsets once UL18 was removed). Because of this, we feel that the data support the conclusion that removal of UL18 from AD169 allows better control by LIR1^+^ NK cells. When HCMV strain Merlin was used, LIR1^+^ NK cells controlled virus spread better than LIR1^−^ NK cells, again independently verifying our previous observations (Fig. 2). Similar to what was observed with AD169, deletion of UL18 from this virus strain also resulted in the elimination of this difference (Fig. 3B). The experiments were repeated using NK cells derived from four independent donors, and in all but one donor the same patterns of results were observed (Fig. 3A and B): donor 302 showed a significant difference between subsets when AD169-ΔUL18 was used (Fig. 3A), while donor 405 showed a small but significant difference between the subsets when Merlin-ΔUL18 was used (Fig. 3B).

The results suggested that UL18 was responsible for the effects on LIR1^+^ and LIR1^−^ NK cell control of viral dissemination and further suggested that UL18 from different strains affected NK cell subsets in a different manner. The crystal structure of the UL18 protein interacting with LIR1 has been determined by Yang et al. (57) and shows that the interactions occur between the α3 domain of UL18 and LIR1 (57). There are three sites of interactions between UL18 and LIR1, compared with two sites between human MHC-I and LIR1, which has been suggested as the reason for a 1,000-fold-higher binding affinity observed between UL18 and LIR1 than MHC-I (57).

To investigate if strain-dependent sequence variability was responsible for the differences observed in LIR1^+^ NK cell control of HCMV dissemination, UL18 sequences from GenBank were identified and the amino acid sequences within the α3 region were aligned (Fig. 3C). In this region, HCMV strains TB40/e and Merlin have a sequence of NKAPDD, while AD169 has DKVPED. These three amino acid differences (N/D, A/V, and D/E) are located adjacent to the additional site of interaction with LIR1 proposed by Yang et al. (57), which results in the increased binding affinity compared to the MHC-I-LIR1 interaction.

Although these amino acids have not been implicated in the direct interaction between UL18 and LIR (57), we hypothesized that they may be of significance because 12 of 13 unique UL18 sequences in the database have either amino acid sequences of NKAPDD or DKVPED (Fig. 3B). We further constructed a neighboring-phylogenic tree to analyze the similarity in the α3 domains among the HCMV viral strains (Fig. 3D). The alignment shows that the two UL18 sequences from TB40/e and Merlin were closely related to each other while the AD169 sequence was located in a different cluster (Fig. 3D). There were no differences between the α1 and α2 regions of UL18 proteins from these three viruses (not shown).

### UL18 sequence variability affects NK cell control of HCMV dissemination.

**FIG 4:**
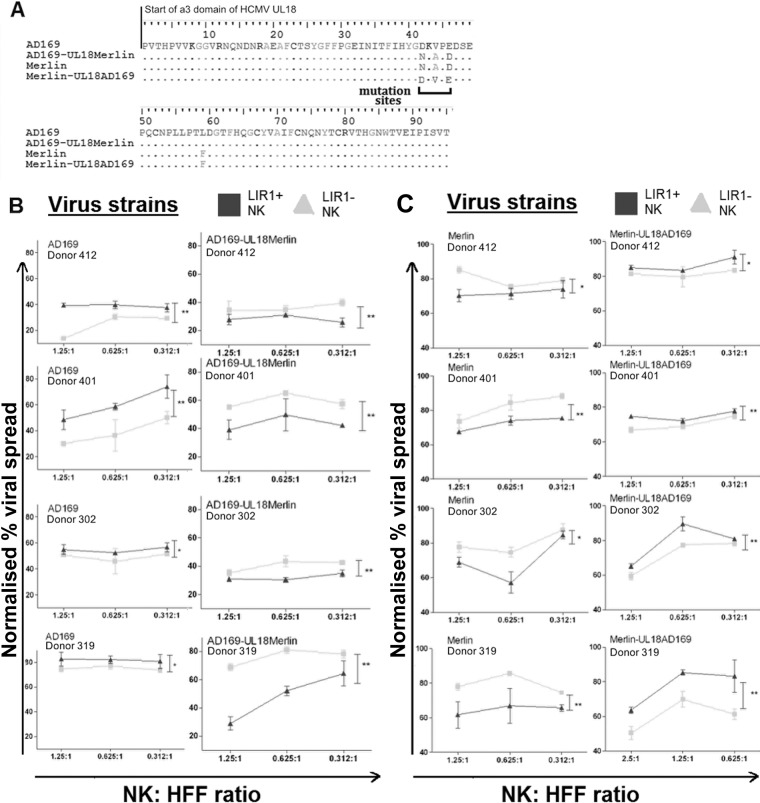
Mutation of HCMV UL18 proteins causes changes in the control of viral dissemination by NK cells. (A) A PCR and sequencing on the α3 region of UL18 was performed to check the mutant viruses AD169-UL18Merlin and Merlin-UL18AD169 and compare them with parental strains. The amino acid alignment is shown with the altered sites indicated. Viral dissemination assays were then carried out using NK cells from four different donors (*n =* 4) cocultured with HFFs infected at an MOI of 0.1 with virus strains AD169 and AD169-UL18Merlin (B) or Merlin and Merlin-UL18AD169 virus (C). AD169- and AD169-UL18Merlin-infected HFFs were stained with IE-Alexa Fluor 488 antibodies before analysis using flow cytometry. Merlin or Merlin-UL18AD169 virus-infected HFFs expressed GFP and did not require additional staining. The NK cell-to-target ratios are 1.25:1, 0.625:1, and 0.312:1. The gray triangles are results from LIR1^−^ NK cells; black squares are the results from LIR^+^ NK cells. Each data point represents 3 independent readouts. The data were analyzed using two-way ANOVA, error bars represent SEM, and significant results (*, *P* < 0.05; **, *P* < 0.01) are indicated.

If the 3 amino acids identified (Fig. 3) were responsible for the differences observed in control of viral dissemination, mutating the UL18 from the sequence of one virus strain to the sequence of the other should reverse the pattern of control observed with LIR1^+^ and LIR1^−^ NK cells. Recombineering was used to mutate the AD169 sequence from DKVPED to the Merlin sequence NKAPDD (AD169-UL18Merlin), a reciprocal mutation was made to the Merlin sequence from NKAPDD to the AD169 sequence DKVPED (Merlin-UL18AD169), and mutant UL18 sequences were verified by PCR amplification and sequencing (Fig. 4A). These mutant viruses were used to infect human fibroblasts, and VDAs were performed using LIR1^+^ and LIR1^−^ NK cells derived from multiple independent donors.

As previously observed, LIR1^+^ NK cells from four different donors were less effective at controlling AD169 dissemination (Fig. 4B, left column). However, when the three amino acids were mutated to the Merlin sequence, the pattern of recognition was changed to that previously seen with Merlin virus, whereby LIR1^+^ NK cells controlled virus spread more effectively than LIR1^−^ NK cells (Fig. 4B, right column). Likewise, as before, LIR1^+^ NK cells from all donors were more effective at controlling Merlin dissemination than LIR1^−^ NK cells (Fig. 4C, left column), and the mutation of three amino acids within UL18 of Merlin to the AD169 sequence reversed this pattern, whereby LIR1^+^ NK cells controlled virus spread less effectively than LIR1^−^ NK cells (Fig. 4C, right column). Together, these results suggest that LIR1-expressing NK cell subsets can differentially control HCMV but that this ability can be altered by variation in the sequence of UL18, the viral ligand to LIR1.

### NKG2C-expressing NK cells influence the functional responses of NK cells but not the dissemination of HCMV.

It has been suggested that receptors other than LIR1 may also influence the activity of NK cells against HCMV-infected cells, in particular the activating receptor NKG2C. Numerous reports have shown a strong correlation between the acquisition of HCMV infection and an increase in the frequency of NKG2C-expressing NK cells in peripheral blood (37–43), and one report has suggested that NKG2C binds with UL18 at very low affinity (36). Although no reports to date have shown differential control between NKG2C^+^ and NKG2C^−^ NK cells of HCMV in standard cytotoxic assays, one recent report does suggest that NKG2Cbright NK cells exhibit higher degranulation against target cells in the presence of serum containing HCMV-specific antibodies (44).

**FIG 5:**
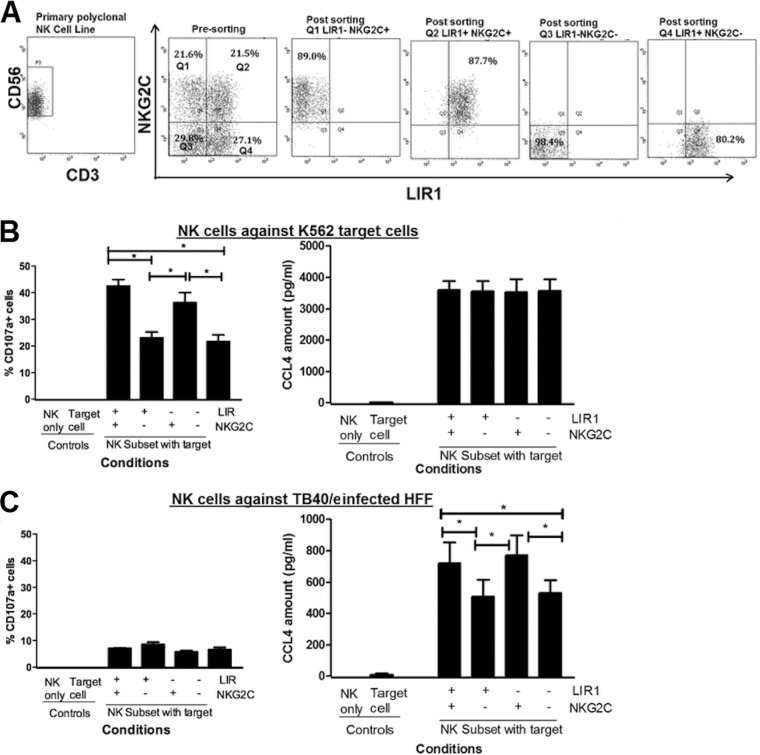
The expression of NKG2C on *in vitro*-expanded NK cells has an effect on NK cell effector functions. (A) *In vitro*-expanded NK cells were stained with anti-CD3, -CD56, -LIR1, and -NKG2C antibodies and sorted by flow cytometry into four subsets based on LIR1 and NKG2C expressions. CD56^+^ CD3^−^ NK cells were first collected (NK) before further sorting into LIR1^−^ NKG2C^+^, LIR1^+^ NKG2C^+^, LIR1^−^ NKG2C^−^, and LIR1^+^ NKG2C^−^. Representative dot plots of the NK cells before and after sorting are shown. Sorted NK cells were then cocultured with K562 targets (B) or HFFs infected with TB40/e at MOI of 5 (C). The NK cell-to-target ratio is 1:1. The percentages of CD107a^+^ cells were measured by flow cytometry after 5 h, and the levels of CCL4 cytokine secretion by NK cells were measured after overnight incubation. NK cell only and target cell only are the control samples. Error bars represent SEM. The experiment was repeated using 3 different donor NK cells (*n =* 3), and the average results were analyzed by the Student *t* test. Significant results (*, *P* < 0.05) are indicated.

We therefore investigated whether NKG2C^+^ NK cells mediated better control of HCMV in our viral dissemination assay. Since we had already shown that LIR1 expression affected NK-mediated control of viral spread, cells were sorted into four subsets based on both LIR1 and NKG2C expression (Fig. 5A). We also analyzed activated NK cell lines with anti-NKG2A, -NKG2C, and -LIR 1 in order to determine the distribution of the inhibitory NK cell receptor NKG2A from four different donors (2 HCMV-seropositive and 2 HCMV-seronegative donors). The results show that there was no or negligible expression of inhibitory receptor NKG2A (HCMV seropositive, 0.4% and 1.1%; HCMV seronegative, 0% and 5%) in cells, and as such it was unlikely to have a significant impact on the functional assay.

To ensure that these subsets maintained their effector functions postsorting, their cytotoxicity (CD107a degranulation) and cytokine secretion against K562 target cells were determined. Both LIR1^+^NKG2C^+^ and LIR1^−^ NKG2C^+^ NK cells degranulated significantly more strongly than the LIR1^+^ NKG2C^−^ and LIR1^−^ NKG2C^−^ NK cells, i.e., NK cell subsets expressing the NKG2C-activating receptor had higher cytotoxicity toward K562 target cells than subsets without NKG2C (Fig. 5B, left); however, expression of LIR1 made little difference in this effector assay. The cytokine release assay showed no significant differences between any NK cell subsets (Fig. 5B, right). Interestingly, these results suggested that different NK cell effector mechanisms could be independently activated.

The four NK cell subsets were also cocultured with strain TB40/e-infected target cells, and CD107a and cytokine responses were measured. All of the subsets displayed low cytotoxicity against TB40/e-infected fibroblasts (Fig. 5C, left), similar to results from previous studies using *in vitro*-expanded NK cell lines (24, 53). In contrast, the cytokine release assay showed that although all subsets maintained their CCL4 production in response to TB40/e-infected fibroblasts, NKG2C+ NK cell subsets had significantly higher CCL4 secretion than did NKG2C^−^ subsets (*P* < 0.05), irrespective of LIR1 expression (Fig. 5C, right). Together, these results suggested that expression of NKG2C enhances CCL4 release by NK cells when interacting with TB40/e-infected target cells but that the induction of cytokine release does not correlate with NK cell degranulation.

**FIG 6:**
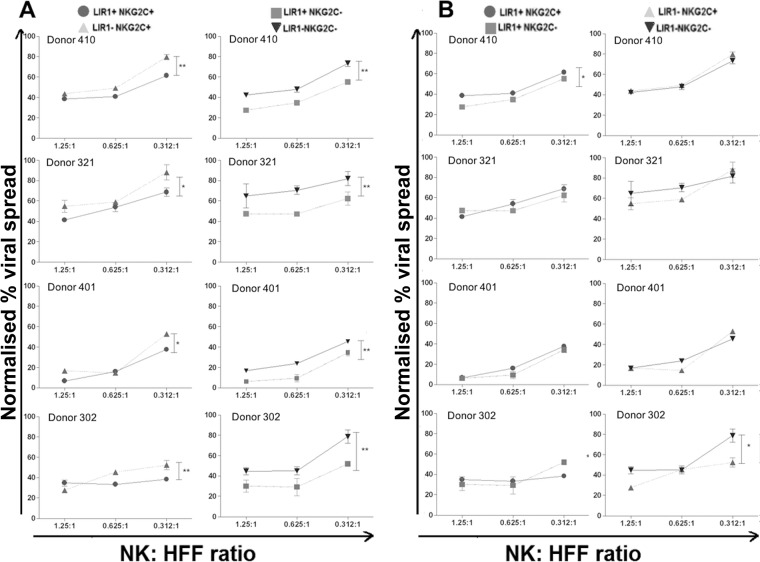
Expression of NKG2C on *in vitro*-expanded NK cells does not control HCMV dissemination more effectively over the long term. Viral dissemination assays were carried out using the NK cells sorted based on LIR1 and NKG2C as previously described and incubated with TB40/e-infected HFFs at an MOI of 0.1. The experiment was repeated four times using NK cells from four different donors. The NK cell-to-target ratios are 1.25:1, 0.625:1, and 0.312:1. The results were analyzed using flow cytometry and then normalized according to uninfected and infected controls. On each graph, the circle represents LIR1^+^ NKG2C^+^ NK cells; the rectangle represents LIR^+^ NKG2C^−^ NK cells; the upward triangle represents LIR1^−^ NKG2C^+^ NK cells; and downward triangle represents LIR1^−^ NKG2C^−^ NK cells. The comparison of LIR1-expressing and LIR1-nonexpressing subsets are shown in panel A, while the comparison of NKG2C-expressing and NKG2C-nonexpressing subsets are shown in panel B. Each data point represents 3 independent readouts. The data were analyzed using two-way ANOVA, error bars represent SEM, and significant results (*, *P* < 0.05; **, *P* < 0.01) are indicated.

The 4 NK subsets were also used simultaneously in a VDA (Fig. 6) using NK cells from four independent donors. In good agreement with our earlier results, NK cell subsets with LIR1 expression, with or without NKG2C, resulted in a lower percentage of viral spread than cells without LIR1 (Fig. 6A). This was observed across all the donors tested. However, when we compared the NKG2C^+^ NK cell subsets against NKG2C^−^ NK cell subsets, with or without LIR1 expression, three of four donors tested showed no difference in the degree of HCMV control (Fig. 6B). In one donor, the LIR1^+^ NKG2C^+^ subset exerted worse control than did the LIR1^+^ NKG2C^−^ NK subset (Fig. 6B, left column), while with another donor, the LIR1^−^ NKG2C^+^ NK subset exerted better control than LIR1^−^ NKG2C^−^ NK cells (Fig. 6B, right column). However, taken together, we concluded that there is no clear difference in the degree of HCMV dissemination when NK cells express or lack NKG2C on their surface.

## DISCUSSION

To date, 10 HCMV gene products and one HCMV microRNA have been shown to interfere with NK cell immune responses, by disrupting both activating and inhibitory signaling to NK cells during HCMV lytic infection (19, 20). Studies into the activity of NK cells *in vitro* against HCMV-infected cells have predominantly focused on effector mechanisms such as cytokine production and cytotoxicity (28, 35). Moreover, although the frequency of NK cells expressing several NK receptors is associated with HCMV serostatus (37, 39, 58), enhancement in NK cell effector functions has been demonstrated only in the presence of anti-HCMV antibodies (41). Currently there is little *in vitro* evidence to suggest that these higher-frequency NK cell subsets confer better control of HCMV in longer-term culture.

A viral dissemination assay that we had previously used to study CD8^+^ T cell responses (51) was established to test NK cell responses against fibroblasts lytically infected with HCMV. This was similar in concept to a recently published focal expansion assay (45) that was used to investigate the control of viral spread by NK cells during HCMV TB40/e infection and the effect of deletion of known viral NK immune evasion genes. This work concluded that NK cells can efficiently control HCMV transmission in different cell types and the UL16 viral protein contributes to the immune evasion of NK cells during HCMV transmission. The focal expansion assay and the viral dissemination assay are new methodological approaches employed for studying the longer-term interaction between NK cells and HCMV-infected cells *in vitro*. Compared with conventional NK cell cytotoxicity and degranulation assays, both focal expansion and viral dissemination assays aim to assess the longer-term control of NK cells on HCMV infection by indirectly measuring changes in the spread of virus in the presence of NK cells. While we did not investigate which NK cell effector mechanisms (either cytokine secretion or direct cell cytotoxicity or both) are effective at limiting growth and dissemination of HCMV in our VDA, it has been demonstrated by Wu et al. (45) that during long-term coincubation of NK cells with HCMV-infected target cells, both direct cell contact and soluble factors like IFN-γ are contributing factors to the control of dissemination (45).

Our assay shows that NK cells can exert effector functions at an E/T ratio as low as 0.3125:1, which is considerably lower and more physiological than results from short-term NK cell cytotoxicity and degranulation assays, which often required an E/T ratio of 10:1 and higher. Our data support the hypothesis that NK cells control low-passage-number strains such as Merlin less effectively than high-passage-number laboratory mutants such as AD169; this is expected, as AD169 is lacking several immune evasion genes (59, 60). TB40/e is known to contain a mixture of virus populations, including some that lack a functional UL141 (an established NK immune evasion gene) (53, 61), and also contains a nonfunctional UL40 gene (62). Thus, it was interesting that the dissemination assay was also able to distinguish between NK cell control of infections with TB40/e and Merlin, underlining the advantages of working with defined strains that express a full complement of HCMV genes when characterizing viral pathogenesis.

Importantly, our assay demonstrated for the first time that the ability of *in vitro*-expanded, activated LIR1^+^ NK cells to inhibit viral dissemination differed from that of LIR1^−^ NK cells and was dependent on natural sequence variation within the ligands expressed by a viral strain. These strain differences are likely due to the differences in the binding kinetics exhibited by UL18 proteins from different HCMV strains to LIR-1 (63, 64). Vales-Gomez et al. demonstrated that a particular isolate of the UL18 protein, variant E, which has amino acid sequences in the α3 region identical to those of strain Merlin UL18, showed a binding affinity that was at least 50-fold lower than the UL18 protein from AD169 (63). They also demonstrated that the UL18 protein of variant E exhibited weaker inhibition against an LIR1-expressing transformed NK cell line than the UL18 derived from AD169 (63). A separate study carried out by Cerboni et al. also showed that purified AD169 UL18 protein was able to inhibit cytotoxicity of the NKL cell line to a greater extent than UL18 protein from a clinical isolate, which again had the same α3 sequence as that found in strain Merlin (64). Interestingly, the three amino acids that we identified as responsible for the differing abilities of NK cells to control dissemination have recently been shown to be under positive selection in HCMV strains, toward the more-inhibitory sequence (65), suggesting that the need to avoid activating NK cells can directly drive virus evolution.

Given the above considerations, it seems likely that UL18 from both the Merlin and TB40/e strains have weaker affinity for LIR1 than for AD169, and as a result LIR1^+^ NK cells received weaker inhibitory signals than from the AD169 strain and were therefore better at controlling HCMV dissemination. It is also possible that UL18 of TB40/e/Merlin also interacts with other NK receptors (leading to enhancement in NK cell function), which have yet to be identified. Alternatively, instead of inducing weaker inhibition signaling via LIR1, the UL18 protein of Merlin/TB40/e might cause a change in clustering of LIR1 and “antagonize” inhibitory signaling. This antagonistic ligand behavior had been suggested in certain peptide-MHC-KIR interactions between HCV and NK cells (66), although the mechanism has yet to be fully established.

Lastly, we examined the effect of expression of the activating receptor NKG2C (67). An increase in the proportion of NKG2C^+^ NK cells is strongly associated with HCMV serostatus (37, 38, 40, 43, 68). However, there is as yet no evidence to suggest that the acquisition of NKG2C on NK cells without the presence of anti-HCMV antibodies confers a stronger immune response against HCMV infection. More recently, another study demonstrated that although HCMV-seropositive patients receiving allogeneic stem cell transplantations have higher proportions of NKG2C^+^ NK cells, there is no obvious change in NKG2C^+^ NK cells between patients with or without HCMV DNAemia 60 days after transplantation (42). In accordance with this, our results showed no differences between NKG2C^+^ and NKG2C^−^ NK cell subsets in their ability to directly control HCMV dissemination (despite NKG2C expression being able to enhance CCL4 cytokine production), suggesting that there may not be a specific functional role for NKG2C in the direct control of HCMV. However, in light of recent published work suggesting that the presence of HCMV-seropositive donor serum can induce stronger responses of NKG2Cbright NK cells in the short-term assay (44), it would be interesting to assess if the donor serum can influence the outcome of these long-term dissemination assays.

In summary, we have presented here an improved, *in vitro* technique of assessing long-term immune control of NK cells against HCMV dissemination. Conventional NK cell cytotoxicity assays focus on the NK cell responses within a few hours of cocultures with target cells. The VDA uses a much lower, more physiological E/T ratio than conventional NK cytotoxicity assays and extends the analysis of NK cell responses to 9 days. Through the viral dissemination assay, we have uncovered new evidence that NK cell subsets respond differently to different variants of viral ligands, but the expression of NKG2C made little difference to the outcome of the long-term HCMV control by NK cells. The VDA could be adapted to analyze other NK cell subsets that have been indicated as “memory-like” and assess whether their *in vitro* control over virus spreading may be similar to that described previously for the murine Ly49H activating receptor and MCMV m157 protein (69). Taken together, these data give the first description of a number of novel interactions between NK cells and HCMV during long-term lytic life cycles. These results may have implications for susceptibility to HCMV infection and to future approaches to vaccination strategies that involve the generation of immunological memory-like responses of NK cells.
